# Topoisomerase IB: a relaxing enzyme for stressed DNA

**DOI:** 10.20517/cdr.2019.106

**Published:** 2020-03-19

**Authors:** Bini Chhetri Soren, Jagadish Babu Dasari, Alessio Ottaviani, Federico Iacovelli, Paola Fiorani

**Affiliations:** ^1^Department of Biology, University of Rome Tor Vergata, Rome 00133, Italy.; ^2^Institute of Translational Pharmacology, National Research Council, Rome 00133, Italy.; ^#^Present address: Department of Research and Application Development, Biogenex Life Sciences, Telangana 501510, India.

**Keywords:** Human DNA topoisomerase IB, mechanism of action, drugs inhibition

## Abstract

DNA topoisomerase I enzymes relieve the torsional strain in DNA; they are essential for fundamental molecular processes such as DNA replication, transcription, recombination, and chromosome condensation; and act by cleaving and then religating DNA strands. Over the past few decades, scientists have focused on the DNA topoisomerases biological functions and established a unique role of Type I DNA topoisomerases in regulating gene expression and DNA chromosome condensation. Moreover, the human enzyme is being investigated as a target for cancer chemotherapy. The active site tyrosine is responsible for initiating two transesterification reactions to cleave and then religate the DNA backbone, allowing the release of superhelical tension. The different steps of the catalytic mechanism are affected by various inhibitors; some of them prevent the interaction between the enzyme and the DNA while others act as poisons, leading to TopI-DNA lesions, breakage of DNA, and eventually cellular death. In this review, our goal is to provide an overview of mechanism of human topoisomerase IB action together with the different types of inhibitors and their effect on the enzyme functionality.

## DNA topoisomerase I

Topoisomerase I (TopI) is a class of enzymes responsible for catalyzing the relaxation of supercoiled DNA during cell essential processes such as DNA replication, transcription, recombination, and chromosome condensation^[1,2]^. TopI enzymes are not dependent on ATP for their catalytic activity, except for reverse gyrase^[3]^. They are divided according to their structure and mechanism of action: Type IA includes bacterial and archaeal TopI^[4]^, topoisomerase III^[5]^, and reverse gyrase, whereas Type IB includes eukaryotic TopI and topoisomerase V^[6]^. Enzymes belonging to family A form a covalent bond between the 5’-end of the broken strand of the DNA and the catalytic tyrosine, instead the family B form the covalent bond between the 3’-end and the catalytic tyrosine. Type IA topoisomerase requires Mg²^+^ for the reaction mechanism and relaxes only the negative supercoils^[7]^, while Type IB appears independent of the Mg²^+^ and relaxes both positive and negative supercoils^[8]^. Members of Type IB family do not share any structural or sequence homology with other topoisomerases and they are functionally diverse from the members of Type IA family^[9]^.

This review focuses on human DNA topoisomerase IB (hTopIB) and its mechanism of action. HTopIB catalyzes the relaxation of supercoiled DNA by cleaving, passing, and religating one strand of the DNA. The catalytic active tyrosine (Tyr723) starts the catalytic process through a nucleophilic attack on the backbone of the DNA that results in the breakage of one of the strands with the enzyme covalently attached to the 3’-phosphate forming the cleavage complex. A second nucleophilic attack occurs after changing the linking number, which is driven by the 5’-hydroxy DNA end, resulting in the restoration of intact double strand DNA. After this step, the enzyme is released^[10,11]^.

The interest in studying hTopIB in recent years arises not only because of their important role in managing DNA topology but also for other major reasons. Foremost is the identification of different topoisomerase-targeting drugs, many of which generate cytotoxic lesions by trapping the enzyme in the covalent complex^[12]^. The other reason is the study of the hTopIB domains that have been published in the past years and provide valuable insights into how these molecular machines function^[13-15]^.

## Domains structure and function

**Figure 1:**
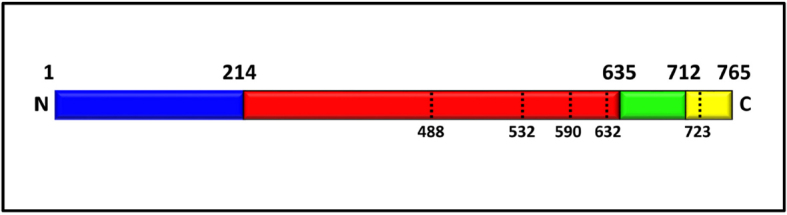
Domain representation of hTopIB showing the catalytic pentad. HTopIB comprises an N-terminal domain (1-214; blue), a core domain (215-635; red), a linker domain (636-712; green), and a C-terminal domain (713-765; yellow). The black lines in the core and C-terminal domain highlight the residues that form the catalytic pentad^[17]^

**Figure 2:**
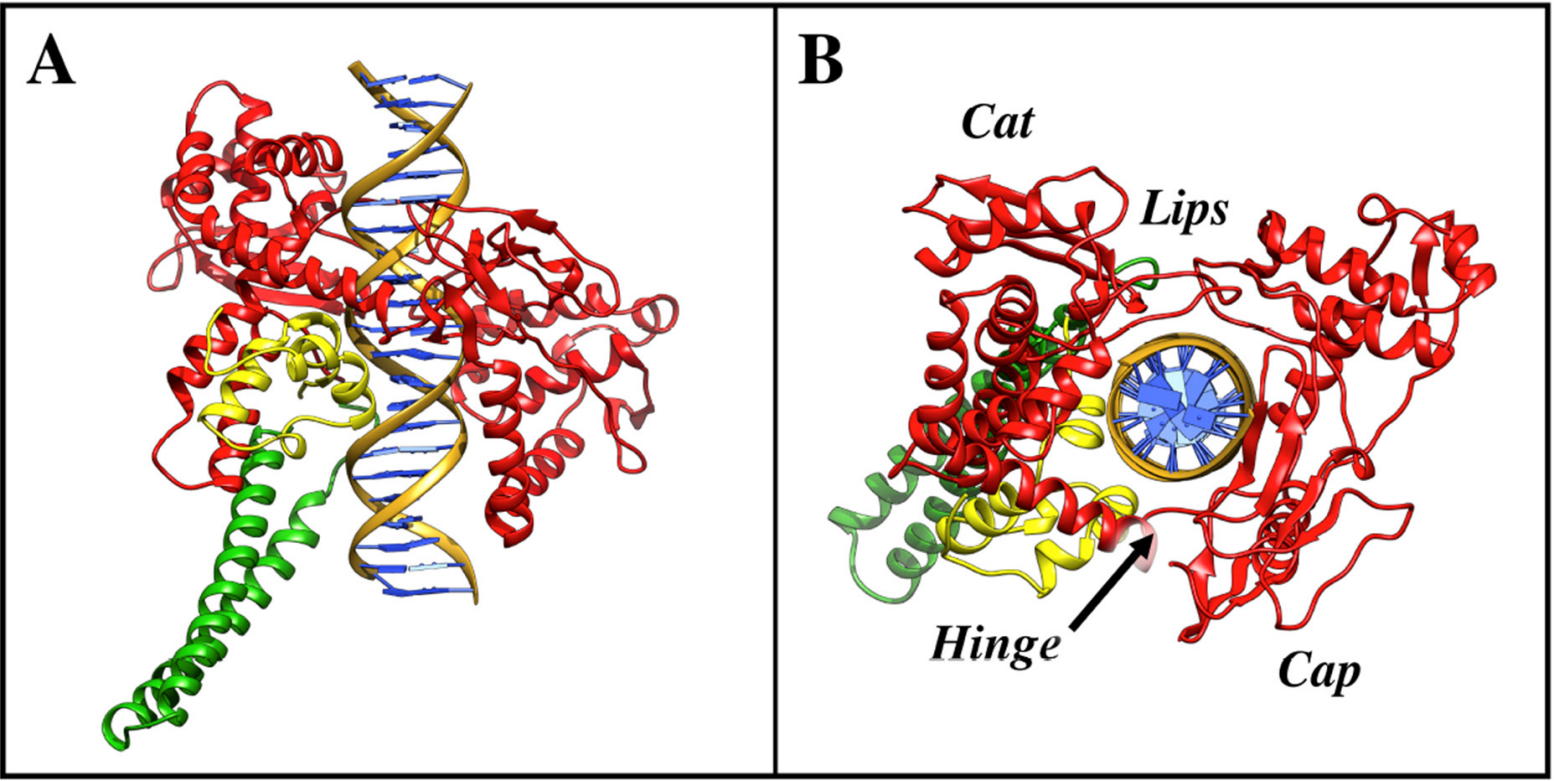
Schematic view of the 3D structure of the non-covalent hTopIB-DNA complex in two different orientations. A: Different domains and DNA forming the complex; B: core subdomains I and II forming the “Cap” region and the residues belonging to core subdomain III and the C-terminal domain forming the “Cat” region. At the top are two loops or lips and opposite to the lips are the chains of the “hinge” residues. This molecular picture was produced with UCSF Chimera program^[35]^

The structure of the hTopIB (PDB ID 1A36) has been resolved based on different studies such as conservation of sequence, sensitivity to limited proteolysis, hydrodynamic properties, and fragment reconstitution experiments^[16]^. These studies indicate that the human enzyme is composed of 765 amino acid residues and subdivided into four distinct domains [Figure 1]: the N-terminal (1-214; represented in blue), the core (215-635; represented in red), the linker (636-712; represented in green), and the C-terminal domain (713-765; represented in yellow)^[14,18]^. The N-terminal domain is 24 kDa and composed of 214 amino acids; it constitutes a highly protease-sensitive, hydrophilic, unstructured region of the hTopIB^[18]^. Crystal structures of different forms of the hTopIB have been determined and show both non-covalent and covalent DNA binding^[16,19,20]^. The crystal structure was obtained with an N-terminal reduced active form of the enzyme in which the first 174 amino acids are missing. However, the X-ray density was only interpretable beginning from residue 215; therefore, the entire N-terminal domain is still not crystallized. Redinbo *et al.*^[20]^ determined the crystal structure of a form of the enzyme where the structure extends back to amino acid residue 203 containing 12 residues of the N-terminal domain. Studies also suggest that residues 191-206 are essential for binding of DNA and processing the enzyme functionality^[21]^. The N-terminal domain is important for catalysis; this theory is also accordant with the study of crystal structure of hTopIB extending to amino acid position 203^[20]^, which shows a close contact between Trp205 and Gly437 of the core domain to permit rotation. The N-terminal domain contains four basic NLSs (nuclear localization signals) and one acidic NLS^[21]^. It serves as the site for interaction with different cellular proteins such as nucleolin, SV40 T-antigen, certain transcription factors, p53, and the WRN protein^[22-24]^. The N-terminal domain is followed by the core domain, which is 54 kDa and is a conserved domain [Figure 1]. The core domain consist of residues 215-635, is involved in catalysis, and is important for the preferential binding of the enzyme to the supercoiled DNA^[25]^. This domain contains all the catalytic residues (Arg488, Lys532, Arg590, and His632) except the active site tyrosine 723 [Figure 1]^[19]^. Based on the structure, this domain is further divided into three subdomains. Subdomains I (215-232 and 320-433) and II (233-319) form a “cap” region containing a pair of a-helices called the “nose cone”. The core subdomain III (434-635) forms the “cat” region [Figure 2]^[26]^. As shown in Figure 2B, opposite to the hinge (which is located at the top of subdomain III) are two loops (called “lips”) that interact with each other by six amino acids and one salt bridge (Lys369-Glu497)^[19]^, to close the enzyme around the DNA. HTopIB clamps around the DNA allowing the interaction of lips through a non-covalent interaction between the carboxylic lateral group of Glu497 and the side-chain amino group of Lys369^[27]^. Opening and closing of the protein clamp during DNA binding and release must involve the breaking of this interaction between the lips and the lifting of the cap away from the base^[16]^. The linker domain is positively charged and incorporates residue 636-712 with a molecular weight of 5 kDa [Figure 1]. The linker, as shown in the three-dimensional structure, is formed by an extended pair of a-helices that protrudes outside the enzyme and connects the core with the C-terminal domain [Figure 2]. This domain is not directly involved in the enzyme catalysis, but it affects hTopIB mechanism of action^[28-30]^. The linker domain has been demonstrated to be in direct contact with the DNA and is one of the most flexible protein regions, as evidenced by multiple non-isomorphous crystal structures and MD simulation. The most important role played by this domain is its contribution in the process of controlled rotation^[31]^. The charged residues on the linker domain and nose cone form an electrostatic interaction with the DNA, allowing a free rotation surrounding the protein^[32]^. The C-terminal domain is an 8-kDa highly conserved domain and contains the catalytically active site Tyr723 [Figure 1]. This domain also contains a number of residues responsible for the interactions with DNA^[33]^. Cleavage of the DNA backbone is performed by nucleophilic attack of Tyr723 on the scissile phosphate; a phosphodiester link is generated between the tyrosine and the 3’ phosphate, releasing a 5’ hydroxyl^[34]^. The C-terminal region has also been shown to bind ATP (adenosine triphosphate) and carry out splicing factor phosphorylating activity^[33]^.

## Mechanics of action and role of the domains in drug sensitivity

**Figure 3:**
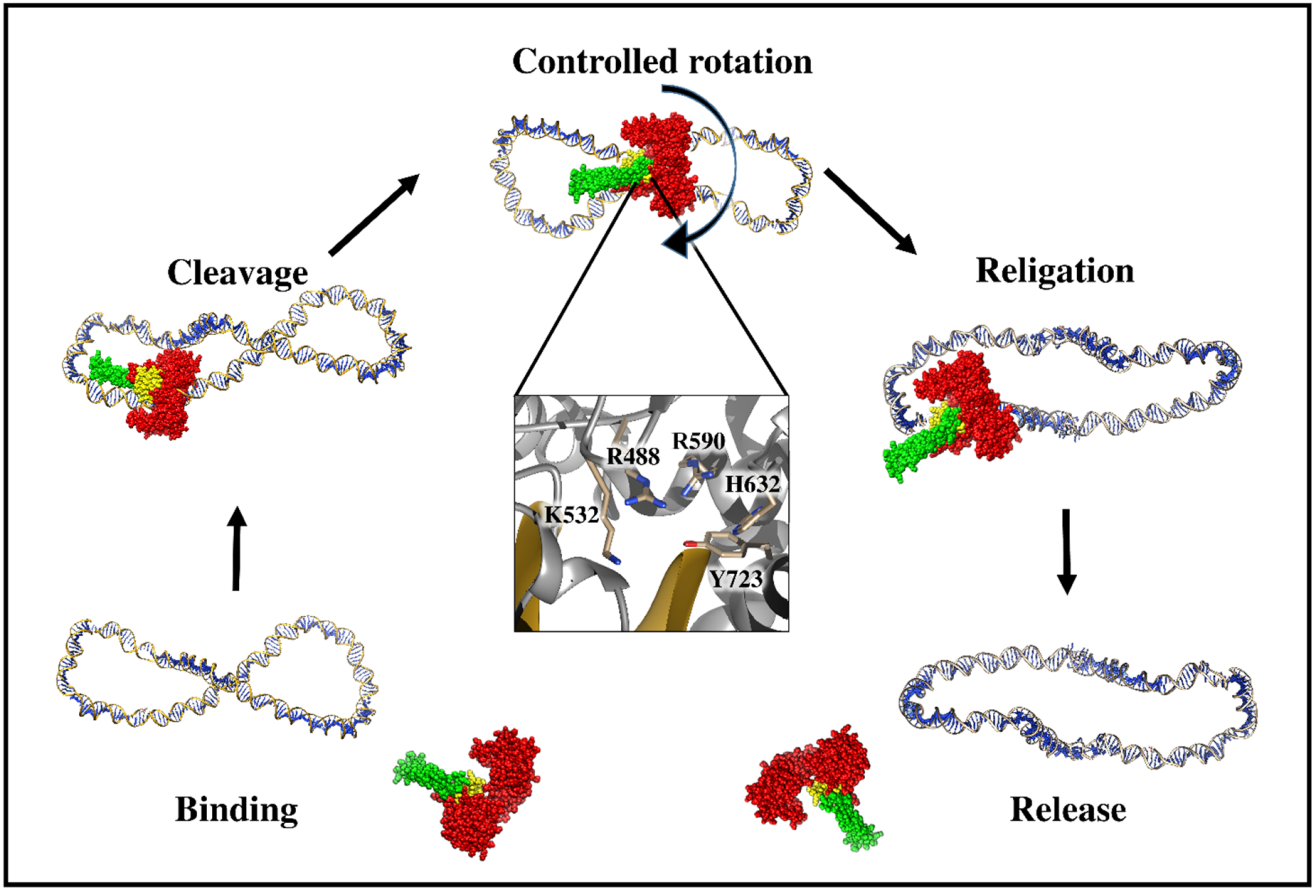
The mechanism of action of hTopIB by controlled rotation. The figure shows the different steps of enzyme catalysis during DNA relaxation, starting from hTopIB binding to enzyme release. The magnification in the controlled rotation step represents the catalytic pentad. The enzyme core domain is in red, the linker is in green, the C-terminal domain is in yellow, and DNA is in blue. This molecular picture was produced with UCSF Chimera program^[35]^

The topoisomerization reaction begins with the binding of hTopIB to the duplex DNA. For the binding to happen, the enzyme should initially exist in an “open” conformation, which is most likely achieved by a hinge-bending motion situated at the edge between core subdomains I and III (residue Pro431) and the boundary between helices 8 and 9 (residue Lys452). This region of the protein is sensitive to proteolysis when the DNA is not present but becomes resistant upon DNA binding. The binding step occurs through the interaction of the charges present on the surface of the enzyme and DNA. This step culminates with the protein that completely embraces the DNA as a “clamp” such that the lips of core subdomains I and III touch each other. As a result of this event, the active site residues are arranged in position for attacking the scissile phosphate, which leads to the cleavage of the strand and formation of the covalent attachment between the enzyme and the 3’ end of the DNA. Once the covalent intermediate has been formed, the release of superhelical tension can occur through one or more cycles of controlled rotation, which involves the ionic charge interactions between the DNA and both the nose cone helices and the linker domain. Consequently, the religation occurs with a nucleophilic attack driven now from the OH’ of the scissile phosphate on the covalent intermediate between the enzyme and the 3’ DNA. Finally, a DNA molecule with a decreased superhelicity is released, hereby allowing the enzyme to undergo another round of DNA binding and relaxation [Figure 3]^[16]^.

**Figure 4:**
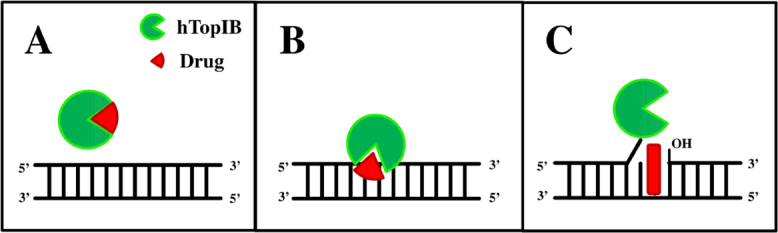
Schematic representation of the different steps of the hTopIB catalysis that can be affected by the inhibitor drugs: inhibition of binding (A); inhibition of cleavage (B); and inhibition of relegation (C). HTopIB is represented in green, while the drug is represented in red

The different steps of the hTopIB catalysis are significant events during the cell cycle since cell vitality can be seriously affected by poisoning these steps. As shown in Figure 4A, the binding event can be perturbed by molecules that can strongly embrace the enzyme impairing its ability to bind the DNA. In the literature, different examples are reported. Erybraedin C (EryC), a natural compound obtained from plant *Bituminaria bituminosa*, was found to inhibit the cleavage in the pre incubation with the enzyme and the compound^[36]^. Et-KuQ, a pentacyclic-diquinoid synthetic compound, was shown to inhibit the cleavage step of the enzyme reaction efficiently with a mechanism similar to EryC^[37]^. Once the cleavage occurs, small molecules are found to target hTopIB by stabilizing the cleavable complex [Figure 4B]. There are many inhibitors of the cleavage step available in the literature but we have to be careful that the inhibition is due to the fact that the drug binds to the enzyme preventing the cleavage. Such points are important to addressed in order to discriminate between the different mechanisms of inhibition. Here, we mention some of the studies where the inhibitor inhibits the cleavage without affecting the binding. Wu *et al.*^[38]^ identified a novel inhibitor that particularly inhibits the cleavage activity of hTopIB instead of allowing the formation of drug-enzyme-DNA covalent ternary complex^[38]^. Another study shows the work on betulinic acid, which inhibits the cleavage kinetics of hTopIB by preventing the formation of apoptotic TopI-DNA complexes^[39]^. The religation step remains the most studied step in the literature because the mechanism behind it is very fascinating. Most of the inhibitors discovered are inhibitors that do not react on the DNA or on the enzyme separately but only after the enzyme cleaves the DNA, transforming the enzyme into a poison [Figure 4C]. Inhibiting the religation phase in a reversible manner is particularly important because the drug affects both healthy and neoplastic cells. The most important and studied metabolite is camptothecin (CPT) and its derivates Irinotecan and Topotecan^[40-44]^. There is another class of inhibitors called Indolocarbazoles, which showed potent non-CPT hTopIB poisons initially but further studies showed that they act on other cellular target besides hTopIB^[45,46]^.

In the last part of this review, we mention some of the most important studies related to the residues affecting drug reactivity. Lisby *et al.*^[47]^ previously demonstrated that deletion of the first 206 amino acids of hTopIB makes the enzyme insensitive towards CPT in the relaxation^[47]^. The study of mutant Glu418Lys showed for the first time the occurrence of different cleavage substrate specificity, thereby confirming the crucial role of core subdomain I in the recognition of the substrate and the requirement of T base at the -1 position of the scissile strand for binding of CPT^[48]^. Chrencik *et al.*^[49]^ suggested that six residues (Phe361, Gly363, Arg364, Glu418, Gly503, and Asp533) present in the Lip1-Lip2 region harbor a CPT resistant mutations cluster^[49]^. Other studies on the Gly363 mutation show the role of this residue in CPT sensitivity^[50,51]^. Even though the linker domain is located far away from the active site of Tyr723, it can deeply affect the drug resistance or sensitivity of hTopIB. The main reason for this long-distance interaction is its flexibility. Evidence of these phenomena comes from several works where key residues involved in the mobility of the domain were mutated. The mutation of Ala653Pro has demonstrated that a large flexibility of the linker domain is associated to CPT resistance^[52]^. This correlation has been shown also in the Glu710Gly mutation^[30]^. This paper demonstrated that a lower degree of linker motion leads to an increase of religation rate and consequently to CPT resistance. On the other hand, in the case of Asp677Gly-Val703Ile double mutant, the reduction of the linker flexibility confers a hypersensitivity to CPT^[53]^. Other evidence of linker involvement in enzyme religation rate comes from the construction of a chimeric enzyme, in which the human enzyme contains a linker from *Plasmodium falciparum* TopI (PfTopI). In the chimeric enzyme, the PfTopI linker shows a great flexibility and confers the chimeric enzyme to be CPT resistant^[54]^. Moreover, inserting a long yeast linker domain in hTopIB drastically altered enzyme function *in vivo*. Expression of this chimera was toxic in yeast even in the absence of CPT, with no particular changes in enzyme catalysis^[55]^. Altering the linker structure, either through changing its flexibility by the mutation of specific residues or by deleting the entire domain, affects the enzyme rate and consequently the CPT reactivity. An additional residue that was shown to impact CPT reactivity is Thr729, which is part of the hydrophobic pocket present in the C-terminal domain. Losasso *et al.*^[56]^ studied this residue extensively by producing different mutations, namely Thr729Ala, Thr729Glu, Thr729Lys, and Thr729Pro, and showed the importance of this residue in modulation of hTopIB DNA binding and drug resistance^[56]^. Residue Asn722, which is present near the Thr729 residue, was also shown to impart CPT resistance to the protein^[57,58]^.

## Conclusion

HTopIB is still a fascinating enzyme that is worth studying. However, some considerations should be given to try to focus the attention of the scientific community. Many inhibitors of the binding and cleavage phases have never entered into clinical protocols. The most important step for inhibition remains the religation and compounds that reversibly inhibit this phase of the enzyme catalysis. The only derivatives of CPT that are now in clinical use targeting hTopIB are still Topotecan and Irinotecan. It is probably time to look for other natural compounds that could inhibit this enzyme. Our group is focusing on a metabolite from an Antarctic sponge that was found to be a potent inhibitor of hTopIB (unpublished results). Perhaps it is time to study and search different ecosystems to obtain promising drugs for cancer therapy, as sometimes it is from nature that we have the best solution.

## Declarations

### Copyright

© The Author(s) 2020.
